# Advances in sequencing technologies for amyotrophic lateral sclerosis research

**DOI:** 10.1186/s13024-022-00593-1

**Published:** 2023-01-13

**Authors:** Evan Udine, Angita Jain, Marka van Blitterswijk

**Affiliations:** 1 Department of Neuroscience, Mayo Clinic, 4500 San Pablo Road S, Jacksonville, FL 32224 USA; 2 Mayo Clinic Graduate School of Biomedical Sciences, 4500 San Pablo Road S, Jacksonville, FL 32224 USA; 3 Center for Clinical and Translational Sciences, Mayo Clinic, 4500 San Pablo Road S, Jacksonville, FL 32224 USA

**Keywords:** Amyotrophic lateral sclerosis, DNA sequencing, Long-read sequencing, SMRT sequencing, Nanopore sequencing, Multi-omics

## Abstract

Amyotrophic lateral sclerosis (ALS) is caused by upper and lower motor neuron loss and has a fairly rapid disease progression, leading to fatality in an average of 2-5 years after symptom onset. Numerous genes have been implicated in this disease; however, many cases remain unexplained. Several technologies are being used to identify regions of interest and investigate candidate genes. Initial approaches to detect ALS genes include, among others, linkage analysis, Sanger sequencing, and genome-wide association studies. More recently, next-generation sequencing methods, such as whole-exome and whole-genome sequencing, have been introduced. While those methods have been particularly useful in discovering new ALS-linked genes, methodological advances are becoming increasingly important, especially given the complex genetics of ALS. Novel sequencing technologies, like long-read sequencing, are beginning to be used to uncover the contribution of repeat expansions and other types of structural variation, which may help explain missing heritability in ALS. In this review, we discuss how popular and/or upcoming methods are being used to discover ALS genes, highlighting emerging long-read sequencing platforms and their role in aiding our understanding of this challenging disease.

## Background

Amyotrophic lateral sclerosis (ALS) is a fatal neuromuscular disease caused by degeneration of both upper and lower motor neurons in the brain, brainstem, and spinal cord, typically displaying accumulation of cytoplasmic TDP-43 [1, 2, 3]. The most common clinical presentations are asymmetric limb weakness, which is seen in about 75% of ALS cases, and bulbar segment onset in about 25% of cases [4]. In addition to the motor symptoms, approximately 60% of patients diagnosed with ALS will experience cognitive and/or behavioral changes, while up to 15% of cases may also receive a diagnosis of frontotemporal dementia (FTD) [5, 6]. Considerable clinical heterogeneity exists in terms of age of disease onset, ranging from 20 to 70 years old [7, 8], and survival after diagnosis, which is generally 2-5 years after onset, with approximately 10% of the patients living for 10 years or more [9, 10]. Diagnosing ALS often proves to be challenging, with the median time of definitive diagnosis between 1 and 4 years to distinguish ALS from other motor neuron diseases (MNDs) [11–13]. Clinical history and physical examination remain the gold standard for diagnosing ALS, even with the advancement of genetic testing [14].

In terms of ALS genetics, approximately 10% of all cases can be classified as familial (fALS), and the remaining 90% of cases are considered sporadic (sALS) [15, 16]. While most fALS cases are caused by mutations in a single gene (monogenic), a subset can be attributed to mutations in several genes (oligogenic) [17, 18]. With a heritability around 60% [19], sALS cases are thought to arise from a combination of variants in many genes (polygenic), probably in addition to environmental factors [17, 18, 20]. It should be noted that dividing ALS into fALS and sALS, although convenient, may not be straightforward. In fact, fALS cases are greatly underreported and can be misclassified as sporadic due to a short disease duration, small pedigrees, genetic heterogeneity, phenotypic variability, and incomplete penetrance [21–24].

Since the discovery of the first ALS gene in 1993, *SOD1* [25], additional genes have been implicated, ranging from causative genes to potential risk factors and disease modifiers (Table 1). Many types of genetic variants may contribute to ALS, such as single nucleotide variants (SNVs) and structural variants. SNVs in coding sequences can be pathogenic missense mutations that lead to the production of proteins with incorrect amino acid sequences, while SNVs in non-coding regions can confer disease risk by affecting the expression or splicing of nearby or distal genes. The other major class of variants, structural variants, encompass large genomic alterations in the form of insertions, deletions, inversions, translocations, repeat expansions, and copy number variations. Structural variants can also occur in non-coding regions of the genome, which often do not change the composition of the mature protein [26], and have been implicated in ALS and FTD (e.g. repeat expansions in *C9orf72*) [27, 28]. Pathogenic mutations in the genes *SOD1*, *C9orf72*, *FUS*, and *TARDBP* are the most frequently observed genetic causes of ALS [25, 27, 29, 30] and comprise both SNVs (i.e. *SOD1*) and structural variants (i.e. *C9orf72*).

**Table 1:** Discovery methods of ALS-associated genes (Adapted from Goutmann et al- Emerging insights into the complex genetics and pathophysiology of amyotrophic lateral sclerosis [31])

Gene by Discovery Method	Chromosomal Position	Inheritance Pattern	ALSoD Category	Pathogenic Mechanism	fALS	sALS	Year of Discovery	References
**Linkage Analysis**								
*ALS2*	2q33.1	AR	Tenuous	Trafficking and degradation of proteins	< 1%	< 1%	2001	[32]
*C9orf72*	9p21.2	AD	Definitive ALS gene	Repeat expansion, Trafficking, degradation of proteins, RNA foci, DPRs	40%	7%	2011	[27, 28]
*DAO*	12q24.11	AD	Moderate evidence	Oxidative stress	< 1%	< 1%	2010	[33]
*DCTN1*	2p13.1	AD, AR	Tenuous	Trafficking and degradation of proteins	< 1%	< 1%	2003	[34]
*ERBB4*	2q34	AD	Moderate evidence	Neuronal cell migration and development	< 1%	< 1%	2013	[35]
*FUS*	16p11.2	AD	Definitive ALS gene	RNA processing/splicing	4%	1%	2009	[29, 36]
*GLT8D1*	3p21.1	AD	Tenuous	Unknown	< 1%	< 1%	2019	[37]
*hnRNPA1*	12q13.13	AD	Definitive ALS gene	RNA processing/splicing	< 1%	< 1%	2013	[38]
*hnRNPA2B1*	7p15.2	AD	Tenuous	RNA processing/splicing	< 1%	< 1%	2013	[38]
*MATR3*	5q31.2	AD	Tenuous	RNA processing/splicing	< 1%	< 1%	2014	[39]
*SETX*	9q34.13	AD	Tenuous	RNA processing/splicing	< 1%	< 1%	1998	[40]
*SOD1*	21q22.11	AD, AR	Definitive ALS gene	Gain of toxic protein	12%	1-2%	1993	[25]
*SPG11*	15q21.11	AR	Tenuous	DNA Repair damage	< 1%	< 1%	2010	[41, 42]
*TARDBP*	1p36.22	AD	Definitive ALS gene	RNA processing/splicing	4%	1%	2008	[30, 43, 44]
*UBQLN2*	Xp11.21	XL	Definitive ALS gene	Trafficking and degradation of proteins	< 1%	< 1%	2011	[45]
*VAPB*	20q13.32	AD	Definitive ALS gene	Trafficking and degradation of proteins	< 1%	< 1%	2004	[46]
**Candidate Gene Analysis**								
*ANG*	14q11.2	AD	Moderate evidence	RNA processing/splicing	< 1%	< 1%	2006	[47]
*ATXN2*	12q24.12	AD	Clinical modifier	RNA processing/splicing	< 1%	< 1%	2010	[48]
*CHMP2B*	3p11.2	AD	Moderate evidence	Trafficking and degradation of proteins	< 1%	< 1%	2006	[49]
*CHRNA3*	15q24	N/A	N/A	Synaptic dysfunction	< 1%	< 1%	2009	[50]
*EWSR1*	22q12.2	N/A	Tenuous	RNA processing/splicing	< 1%	< 1%	2012	[51]
*FIG4*	6q21	AD	Moderate evidence	Trafficking and degradation of proteins	< 1%	< 1%	2009	[52]
*GLE1*	9q34.11	N/A	Moderate evidence	RNA processing/splicing	N/A	N/A	2016	[53]
*NEFH*	22q12.2	AD, AR	Tenuous	Trafficking and degradation of proteins	< 1%	< 1%	1999	[54]
*PON1-3*	7q21.3	N/A	Tenuous	Lipid metabolism	< 1%	< 1%	2009	[55]
*PRPH*	12q13.12	AD, AR	Tenuous	Trafficking and degradation of proteins	< 1%	< 1%	2004	[56]
*SIGMAR1*	9p13.3	AR	Tenuous	Trafficking and degradation of proteins	< 1%	< 1%	2011	[49]
*SQSTM1*	5q35.3	AD	Tenuous	Trafficking and degradation of proteins	~ 1%	< 1%	2011	[57]
*TAF15*	17q12	N/A	Tenuous	RNA processing/splicing	N/A	N/A	2011	[58]
**GWAS**								
*CFAP410 (C21orf2)*	21q22.3	N/A	Strong evidence	Cytoskeletal defects	< 1%	< 1%	2016	[59]
*CAMTA1*	1p36.31-p36.23	AD	Clinical modifier	Trafficking and degradation of proteins	N/A	N/A	2016	[60]
*CCNF*	16p13.3	AD	Strong evidence		~ 1-3.3%	< 1%	2016	[59]
*KIF5A*	12q13.3	AD	Definitive ALS gene	Axonal pathology	N/A	N/A	2018	[61]
*NIPA1*	15q11.2	AD	Strong evidence	Repeat expansion	N/A	N/A	2019	[62]
SARM1	17q11.2	N/A	Moderate evidence	Axonal pathology	N/A	N/A	2021	[63]
*TIA1*	2p13.3	AD	Tenuous	Oxidative stress	~ 2.2%	< 1%	2017	[64]
*UNC13A*	19p13.11	N/A	Definitive ALS gene	Synaptic dysfunction	N/A	N/A	2012	[65, 66]
**WES/WGS**								
*ANXA11*	10q22.3	AD	Definitive ALS gene	Trafficking and degradation of proteins	~ 1%	~ 1.7%	2017	[67]
*CHCHD10*	22q11.23	AD	Definitive ALS gene	Trafficking and degradation of proteins (Mitochondria)	< 1%	< 1%	2014	[68, 69]
*DNAJC7*	17q21.2	N/A	Moderate evidence	Trafficking and degradation of proteins	< 1%	< 1%	2019	[70]
*NEK1*	4q33	AD	Definitive ALS gene	Trafficking and degradation of proteins	~ 1-2%	< 1%	2015	[71–74]
*PFN1*	17p13.2	AD	Definitive ALS gene	Trafficking and degradation of proteins	< 1%	< 1%	2012	[75]
*TBK1*	12q14.2	AD	Definitive ALS gene	Trafficking and degradation of proteins	~ 3%	< 1%	2015	[76–78]
*TUBA4A*	2q35	AD	Strong evidence	Trafficking and degradation of proteins	< 1%	< 1%	2014	[79, 80]
*VCP*	9p13.3	AD	Definitive ALS gene	Trafficking and degradation of proteins	1%	1%	2010	[81]
*ERLIN2* Homozygosity mapping	10q24.31	AR	Tenuous	Lipid transport	N/A	N/A	2012	[82]
*EPHA4* Functional study	2q36.1	N/A	Definitive ALS gene	Axonal pathology	N/A	N/A	2012	[83]
*OPTN* Homozygosity mapping	10p13	AD, AR	Definitive ALS gene	Trafficking and degradation of proteins	1%	< 1%	2010	[84]
*VEGFA* Functional testing	6p21.1	N/A	Tenuous	Angiogenesis	N/A	N/A	2009	[85]

*AD* Autosomal dominant inheritance, *AR* Autosomal recessive inheritance, *XL* X-linked inheritance. ALS Online Database (ALSoD) category definitions = *Definitive ALS gene* Variants in these genes show increase in risk of ALS based on statistical test, *Clinical modifier* Variants in these genes have been linked to a difference in clinical phenotype of ALS, *Strong evidence* Variants in these genes have shown to increase risk in well-conducted recent studies but require replication or resolution of conflicting evidence, *Moderate evidence* Variants in these genes have been associated with ALS in smaller studies or there may be contradictory evidence, *Tenuous* Variants in these genes have been associated with ALS in smaller studies a while ago but have not stood up to replication. They may also be genes that have been associated with ALS-like diseases, which are no longer recognized as a clinical diagnosis of ALS. *N/A* Information is not available. fALS represents the reported frequency of these variants in familial ALS cases and sALS represents the reported frequency of these variants in sporadic ALS cases

Early genetic studies of ALS relied on mapping a chromosomal location in ALS pedigrees to nominate disease genes using a method called DNA linkage analysis. One of the most notable examples is the identification of chromosomal region 9p21 [86–90] and its subsequent refinement [91–94], which eventually led to the groundbreaking discovery of a repeat expansion in the gene *C9orf72* [27, 28]. Another popular method is a genome-wide association study (GWAS). The goal of a GWAS usually is to quantify differences in allele frequencies across the genome between cases and controls. It is an unbiased approach to identify disease-associated common genetic variants. This is often performed by genotyping numerous single nucleotide polymorphisms (SNPs), which are SNVs that are present in at least 1% of the population [95], using various methods (e.g. microarrays). A GWAS can reveal significant associations; please note, however, that association does not equal causation. In the ALS field, GWAS has resulted in several discoveries, including that of *UNC13A* and *KIF5A* [61, 91]. Thus far, GWAS has only explained a small proportion of genetic susceptibility to ALS, suggesting rare and structural variants may account for a substantial proportion of missing heritability [96].

## Sequencing methods

### Sanger sequencing

The methods described above were used to discover disease-associated genomic loci and genes, but, on their own, are unable to provide sequence information about the genes themselves. For that, sequencing methods could be used. Sanger sequencing was one of the earliest sequencing methods developed to determine the DNA sequence. Nowadays, a modified version is used, where genomic DNA is amplified using primers that target a region of interest. Subsequently, the amplicon is sequenced by capillary electrophoresis. Sanger sequencing is a reliable method with up to 99.9% accuracy. Currently, in the ALS field, it is most commonly used to screen samples for mutations in well-known ALS genes [97] and to confirm the presence of mutations identified through other methods, such as "next-generation" sequencing [98].

### Next-generation sequencing

More recently, sequencing efforts have shifted toward next-generation sequencing techniques, such as whole-exome sequencing (WES) and whole-genome sequencing (WGS). This enabled researchers to continue their search for ALS-linked genes, even in cases without a multigenerational family history and in families with limited DNA sample availability. These technologies leverage high-throughput, large-scale parallel DNA sequencing of all coding sequences (WES) or the entire genome (WGS) and can be very powerful in addressing monogenic disorders [99–101]. Briefly, to perform next-generation sequencing, DNA must be fragmented, regularly by shearing, sonication, or enzyme digestion. Then, linkers or specialized adaptors are often added at the ends of the fragmented molecules to create template libraries. The resulting clusters of DNA fragments are typically amplified on a chip, producing millions of copies of double-stranded DNA. Frequently, a signal for each base is detected using fluorescence during the sequencing procedure. Using this method, it is possible to produce either single (one direction of sequencing) or paired (both directions of sequencing) end reads that will need to be analyzed by a process known as base calling. Different software programs are used to sort and align DNA sequences to the reference genome and analyze the data efficiently [102]. As the cost of sequencing is declining, bigger cohorts are being sequenced. This enables the identification of coding and non-coding variants associated with ALS. To prioritize variants, analyses like unsupervised learning [103, 104], linear mixed-modelling, and gene burden testing [105] have been employed. For example, an exome-wide rare variant burden analysis confirmed the significant GWAS hit in *KIF5A*, and additionally, revealed significant associations for *TBK1* and *NEK1* [71, 76, 106]. To extend aforementioned studies, Project MinE (see section ‘Collaborative sequencing efforts’ for additional details) aims at performing WGS on 15,000 ALS patients and 7500 matched controls. In addition to *KIF5A* and *NEK1*, this project already identified *CFAP410* [59, 61, 71, 107], as well as detected structural variants in *C9orf72*, *VCP*, and *ERBB4* [108]. Nonetheless, WES and WGS are not entirely without limitations. One major drawback of WES is its restriction to exonic regions, generally missing intronic, promoter, and enhancer variants. Both WES and WGS depend on read quality and sequencing depth, and additionally, they encounter issues calling structural variation. For example, the intronic repeat in *C9orf72* has been identified though linkage mapping and the locus has been implicated in several GWASs; however, the expansion is challenging/arduous to capture and size by WGS because of the difficulty aligning short-read data of microsatellite and minisatellite DNA sequences [109, 110]. Though bioinformatic tools have now been developed to detect structural variation in short-read data (ExpansionHunter, HipSTR, GangSTR, etc.) [102, 111, 112], other approaches that potentially provide a more in-depth characterization will be valuable in understanding the missing heritability in ALS and/or identifying disease modifiers [113].

### Long-read sequencing

**Fig. 1:**
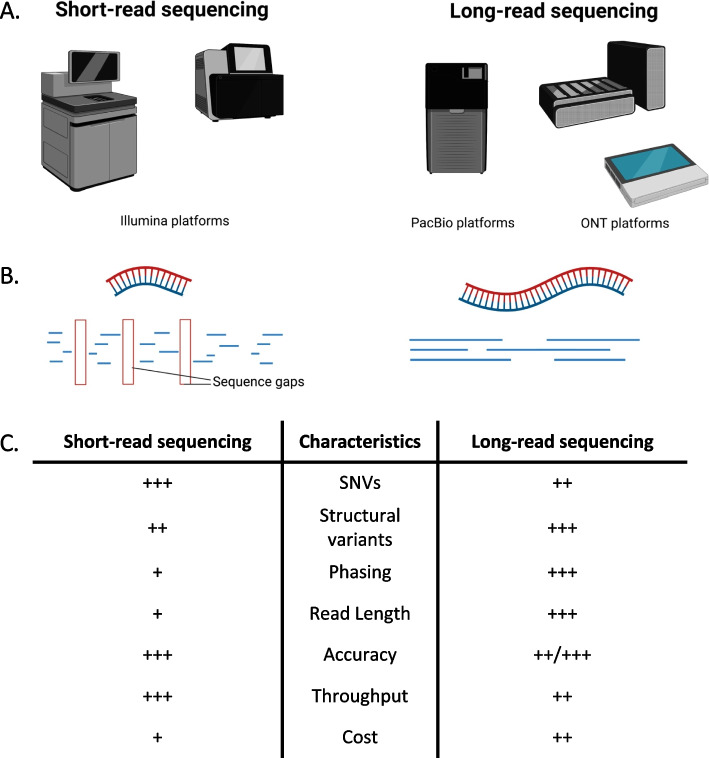
Overview of short-read and long-read sequencing technologies. **A** Examples of widely used platforms for short-read and long-read sequencing technologies. **B** The primary difference between short-read and long-read sequencing technologies is the significant increase in read length. In contrast to short-read sequencing (150–300 bp), long-read sequencing has the capacity to sequence reads spanning multiple kilobases in one single read, thereby requiring fewer reads to cover the same gene. The read overlap seen with long-read data reduces the sequence gaps as observed in short-read data. **C** Semi-quantitative comparison of short-read and long-read sequencing of various features including the ability to detect single nucleotide variants (SNVs), structural variants, and complete genome phasing, as well as the overall read length, accuracy, throughput, and sequencing cost

While the previously discussed approaches have been crucial to gene discovery in ALS, newer technologies are beginning to take precedent to address outstanding genomic questions. One approach that is continuing to gain popularity is long-read sequencing. Though there are multiple platforms available, and others in development, long-read sequencing can broadly be defined as any single-molecule sequencing approach that is capable of generating reads that are multiple kilobases in length. Platforms from Pacific Biosciences (PacBio) [114, 115] and Oxford Nanopore Technologies (ONT) [116, 117] appear to have emerged as the leading long-read sequencing technologies (Fig. 1).

PacBio has developed a technology called single-molecule real-time (SMRT) sequencing [114, 118]. Originally developed in 2009, PacBio’s SMRT sequencing produces long reads by incorporating phospholinked nucleotides labelled with different colored fluorophores. SMRT sequencing is achieved at zero mode waveguides (ZMWs), which are tiny wells with a glass bottom, that can hold a single DNA molecule. PacBio’s SMRT cells accommodate millions of these ZMWs for sequencing to occur. At each ZMW, an anchored DNA polymerase will incorporate a labelled nucleotide, complementary to that from the template DNA molecule. When this occurs, light is emitted and the signal, which is unique to each base, is measured in real time [114, 118, 119]. More recently, PacBio has implemented updated sequencing technologies with the Sequel II system, and in 2019 they introduced high fidelity (HiFi) sequencing, which drastically improves the accuracy of the sequencing by utilizing circularized adapters (SMRT Bell Adapters) so that each molecule can be sequenced multiple times [120]. With this option, the user can computationally call a consensus sequence – a circular consensus sequence (ccs read) – to obtain the most accurate read possible, with reads being > 99.9% accurate [120]. While HiFi sequencing may be the best option for obtaining the most accurate sequences, another method, called continuous long-read sequencing (clr) generates longer reads. Additionally, in 2022 PacBio announced a new platform called the Revio. The Revio is a sequencer that performs HiFi sequencing at a much greater scale than what was previously achievable. It is able to generate data at a 15x higher throughput than the Sequel II, containing 25 million ZMWs in a single SMRT cell [121].

The other most prominent long-read sequencing technology is from ONT. ONT has pioneered the nanopore technology for long-read sequencing, where they are able to sequence extremely long reads while also yielding many reads [116, 122]. Nanopore sequencing works by using a motor protein and tether to pull a single-stranded DNA molecule through a nanopore. The change in ionic current is measured as each nucleotide is passed through the nanopore, with unique signals for each base [116, 123]. There are three main ONT sequencing platforms, including the MinION [116, 122, 124–126], GridION [127, 128], and PromethION [129, 130], which are different sequencers with the same underlying technology, but varying strengths and weaknesses. The MinION is the smallest and most cost effective of the machines offering desktop and portable sequencing options, but has the lowest yield, lowest accuracy (initial estimates around 60%) and can only sequence one flow cell at a time [116]. The GridION allows for up to five flow cells at a time and can generate 250 Gb of sequencing data but does not offer much improvement from the MinION other than scalability [131]. Lastly, the PromethION offers up to 48 flow cells and produces the most accurate sequencing data offered by ONT, with a read accuracy of up to 99% [132]. Overall, considerations of cost, read length, read depth, and sequencing accuracy need to be considered when choosing which long-read sequencing technology to use.

### Long-read sequencing applications

Long-read sequencing has been used in other fields to create reference genomes and/or transcriptomes for a diverse number of species [122, 133–136]. More recently, in humans, long-read WGS has been utilized by the telomere-to-telomere consortium to sequence the first “complete human genome” [137, 138]. This expedition begun to build a reference genome without any gaps in humans, where researchers used both PacBio and ONT to sequence every part of the genome, including the telomeres and centromeres that were previously too difficult to capture [137, 138]. One of the main advantages of long-read WGS is its ability to cover these kinds of complex genomic regions and find structural variation [139–142]. Structural variation, including insertions, deletions, inversions, translocations, expansions, and copy number variations are difficult to capture with short-read sequencing because the length of each sequencing read is often shorter than the size of the structural variant [140, 141, 143]. As previously mentioned, (see Background), structural variation may explain some of the missing heritability in ALS [113]. Thus far to our knowledge, only one published study has performed long-read WGS in the context of ALS, where they focused on *C9orf72* repeat expansions [109]. Using the ONT MinION, they could not detect any reads covering the *C9orf72* expansion, while with PacBio SMRT sequencing there was 8x coverage of the expansion [109]. Currently, no large-scale association studies in ALS have been reported (yet) with long-read WGS. Studies have utilized long reads, however, to identify many structural variants in a small number of subjects [144], and been used to resolve complex regions that harbor known polymorphisms [145–148] or to validate structural variants that have been determined by other methods [149, 150]. Therefore, there is great promise for this technology to be used in the future of ALS research.

Rather than performing genome-wide long-read sequencing, targeted sequencing approaches can be used to scrutinize highly complex regions of the genome where there is known genetic risk. For very long variants or repeat expansions, WGS may not have enough reads to sufficiently cover those regions [109]. Therefore, targeted methods are extremely useful for understanding repeat expansions and have been applied in many neurological diseases, such as those associated with repeat expansions in *FMR1* [151, 152], *NOTCH2NLC* [153], *DM1* [154] and *HTT* [155] to name a few. PacBio and ONT both offer targeted sequencing platforms that select a specific region of the genome using primers, probes, or CRISPR-based methods. PacBio’s targeted sequencing method, No-Amp (no amplification) sequencing is a DNA sequencing approach that can be used with CRISPR-Cas9 and custom designed guide RNAs to target a specific region in the genome [156]. The main advantages of this approach over alternative methods are that it can measure the exact length and sequence of the expansion, while detecting DNA methylation (as can ONT) [156]. In the design of No-Amp studies, researchers can elect to capture the flanking regions around the expansion so that expansion length, which may act as a disease modifier in certain diseases, can be accurately sized by ensuring the entire expanded region is captured. This has been applied, for example, to sequence through the *C9orf72* repeat expansion [109, 157]. No-Amp sequencing that has been completed in this region has demonstrated that the expansion length from No-Amp is correlated with the estimated length from Southern blotting, the current gold standard for sizing the *C9orf72* expansion [157]. Targeted sequencing has been done for other ALS genes, where long-read sequencing revealed an unstable intronic repeat with variation in the sequence of the gene *WDR7,* which was missed by other sequencing technologies [158]. No-Amp sequencing can be done on a number of genes at the same time, especially with smaller expansions. This multi-gene approach has been used to look at repetitive regions that cause various spinocerebellar ataxias (SCAs) [159], such as SCA1, SCA2, SCA10, and SCA36, as well as myotonic dystrophy type 1, where it was possible to size the repeats and detect interruptions in the sequence within the each repeat [159, 160]. One of these diseases, SCA2 is caused by a repeat expansion in the gene *ATXN2* [161], which has been demonstrated to be a genetic modifier of ALS [48, 162, 163].

Here, we have highlighted the power of long-read DNA sequencing (Fig. 1), specifically demonstrating its ability to sequence through highly complex regions of the genome [141]. As mentioned previously, the two technologies highlighted above both detect DNA methylation, however, tools for analyzing this data are in earlier stages of development [164, 165]. In addition to these two main platforms, other options from companies such as Beijing Genomics Institute, 10x genomics, and Illumina are available or in development. Alternatively, non-sequencing, optical mapping approaches from Bionano and OpGen can be used to visualize large chromosomal abnormalities. Despite the many advantages of long-read sequencing, there remain limitations. Primarily, it is generally more expensive than alternative approaches, while generating fewer reads than short-read sequencing [166]. Additionally, there is a great computational cost. Data files can be on the scale of terabytes of data per flow cell, which makes data storage and processing costly. Moreover, the quality of the material required to guarantee sequencing integrity can be a challenge when working with frozen tissue, particularly tissue from the central nervous system. Finally, though much longer reads can be obtained than with traditional sequencing methods, it is inevitable that some structural variants will exceed read length capabilities. Nevertheless, this technology is continuing to advance, with reduction of cost and rapid improvements to the number, the length, and the accuracy of reads that are generated.

### Multi-omics

Thus far, we have focused on the use of single DNA sequencing techniques to identify causal variants and genes, as well as genetic modifiers and/or risk factors of ALS. While these approaches have and will continue to be widely useful, there is tremendous value in integrating multiple data types to further prioritize disease-relevant or causative genes. Functional genomic and/or multi-omic approaches rely on incorporating DNA sequencing data with other data types to look at the epigenome, transcriptome, proteome, etc. Methods for these analyses are very powerful for highly polygenic diseases, where multiple common variants may confer some disease risk if not sufficient to cause disease. Given the apparent polygenic nature of sALS [61, 107], it will be important to use these integrative approaches to nominate genes that may be impacted by identified genetic variants. Herein, this review will discuss common functional genomic/multi-omic approaches while highlighting how they have been used in ALS or related diseases.

Perhaps the most commonly used approach in multi-omic research is bulk short-read RNA sequencing (RNAseq). RNAseq is a next-generation sequencing method that can be used to quantify gene expression and splicing for many genes across the entire transcriptome. RNAseq analyses are commonly used in animal and cell models of ALS to determine the transcriptomic effects of gene knockout or overexpression [167–171]. Standard disease-relevant RNAseq analyses in humans are used to perform case vs. control analyses to identify differential gene expression and differential splicing across the transcriptome. Other analyses, like network and pathway analyses can be done to find networks of genes whose expression is correlated and determine the dysregulated molecular pathways, rather than single genes. This has been done many times across neurodegenerative diseases including in the ALS field [172–175]. Differential expression analysis of human brain tissue in the context of ALS, for instance, has revealed that sALS and *C9orf72*-linked ALS demonstrate wide-spread splicing alterations, but have unique transcriptomic profiles [172, 174], and later showed that repetitive elements are increased in *C9orf72*-linked ALS [167, 173]. Another RNAseq study in ALS identified three major unique molecular subtypes - retrotransposon activation, oxidative damage, and glial activation - of ALS, based on unique transcriptomic profiles [175]. Additional RNAseq data was generated in human ALS tissue, which was used to show truncated transcripts of *STMN2,* a microtubule gene that has been implicated in ALS and FTD, are present specifically in tissues with TDP-43 pathology [176–178]. Further analyses of RNAseq data revealed the mechanism by which ALS-associated SNPs in the gene *UNC13A* are likely pathogenic [65, 91, 107]. Moreover, RNAseq was performed in multiple cell types and in human tissues to show that variants in *UNC13A* increase the inclusion of a cryptic exon, which is an exon that is present within a normally intronic region and is incorrectly included in the mature mRNA, possibly by preventing TDP-43 from binding to the cryptic splice site [179, 180]. These cryptic exons may be particularly relevant in ALS, as one of the roles of TDP-43 is to prevent their inclusion into mature RNAs [167]. Cryptic splicing events may continue to be observed in additional genes relevant to ALS and are proposed to be pathogenic by either introducing an early stop codon, causing a loss of expression, or by being incorporated into the mature RNA, and thus potentially leading to the production of a toxic protein. Future studies, like the ones described here, are essential for increasing our understanding about how genetic variants may confer pathogenicity [167].

Currently, single-cell and single-nuclei RNAseq approaches are being used to identify cell type changes and transcriptomic alterations within specific cell types. Many single-cell studies have been utilized in the cancer field and in other neurodegenerative diseases, like Alzheimer’s disease [181]. More recently, researchers have begun to perform single-cell sequencing in the ALS field. These studies have pointed toward alterations in multiple cell types, suggesting that genetic risk of ALS is conferred through interneurons, motor neurons/Betz cells, and oligodendrocytes [182]. This goes beyond bulk RNAseq methods, allowing researchers to find cell type alterations that are unable to be detected with current pathological measures.

Other newer approaches can be used to pinpoint genes and proteins that change in specific regions of a cell or tissue. Spatial transcriptomics has been used more widely in cancer and tumor biology, with more limited applications in neurodegeneration and ALS [183, 184]. One study performed spatial transcriptomics in mouse and human ALS tissue and found alterations in microglia and astrocyte dynamics in the spinal cord [183]. Another study found 16 transcripts that were dysregulated in the granular cell layer of ALS spinal cords [184]. This approach can be further applied to look for transcriptomic changes surrounding the various pathological features of ALS (i.e. TDP-43), as has been done in the context of amyloid pathology in Alzheimer’s disease [185].

In addition to these short-read RNAseq approaches, long-read RNAseq can also be used to improve upon short-read approaches by detecting more alternative splicing events than short-read sequencing [186] and identifying novel transcript variants and genes, which may be particularly relevant to ALS given the strong implication of RNA-binding proteins in disease pathogenesis [187–192]. PacBio [159] and ONT [193] also dominate the long-read RNAseq field with RNAseq possible on all the previously described platforms. Efforts are currently ongoing to apply long-read RNA sequencing to sizeable human datasets, and thus far have primarily been used for transcriptome reference assembly.

Multi-omic approaches, however, are not just limited to expression profiling. Other approaches, such as ATAC-seq (chromatin accessibility) [194], CHIP-seq (protein-DNA/RNA binding) [195], and HI-C (genome structure/interactions) [196] can be used to look at regulatory changes across the entire genome. Relevant to ALS, the Answer ALS consortium [197] is pioneering efforts to integrate many types of multi-omic data, including genomic, transcriptomic, epigenomic, proteomic, and metabolomic data, with the end goal of developing a cure for ALS (see section ‘Collaborative sequencing efforts’ for additional details). Various multi-omic studies relevant to ALS have been completed in induced pluripotent stem cell (iPSC) models [198, 199], with one group integrating ALS GWAS with RNAseq, ATAC-seq, CHIP-seq, and HI-C to identify *KANK1* as an ALS risk gene [199].

A common way to integrate multi-omic data is through quantitative trait loci mapping (QTLs) [200]. QTLs can be used to determine the molecular effect of a genetic variant, where the presence of a variant can be associated with expression (eQTL), splicing (sQTL), methylation (meQTL), or other -omic measures. QTL results are used in transcriptome-wide association studies (TWAS) to nominate genes that may be impacted by disease-associated genetic variants [201]. Two TWASs have been completed in ALS, where expression was estimated from human brain tissue and blood. These studies have been able to validate previously identified GWAS loci, and nominate 7 and 5 novel genes, respectively [202, 203]. Like loci identified through GWAS and other methods, TWAS results require validation and replication, however it is clear that TWAS itself can also be used to identify novel loci.

## Collaborative sequencing efforts

In many fields, not just in the ALS community, efforts are being made to generate large datasets that include many individuals in order to increase power to detect genetic variants. Project MinE aims at generating WGS from greater than 15,000 ALS patients and an additional 7500 controls [204]. The Clinical Research in ALS and related disorders for Therapeutic development (CReATe) consortium seeks to discover ALS biomarkers, creating a data repository that contains WGS and biospecimens for > 1000 subjects with ALS or other MNDs [205]. Answer ALS [197], which was also mentioned earlier, is focused on developing ALS patient-derived cell lines and generating multi-omic data from cell lines and human tissue data. NeuroLINCS, which is a major contributor to Answer ALS, is a collaborative effort to perform multi-omic profiling of iPSC-derived motor neurons [198]. Likewise, the NYGC ALS Consortium, which contributes to the sequencing effort of Answer ALS, is working to integrate WGS and RNAseq data from human ALS tissue [61]. Each of these consortia and collaborative efforts have the same end goal – to work towards developing a treatment for ALS. It should be noted, though, that these efforts are all works in progress and do not provide all answers to the many challenging questions they are attempting to address. Of course, creating these large datasets will yield many new lines of investigation. However, researchers should be mindful that like other studies, validation and replication remains crucial.

These collaborative efforts use many of the previously discussed methods and technologies and apply them to global datasets to identify disease-relevant variants and genes that we currently do not have enough power to detect. Analysis of these datasets may require machine learning or deep learning approaches with the goal of deciphering causal variants and identifying disease subtypes that may contain distinct genetic drivers [206, 207]. These datasets seek to move/ will potentially move the field towards developing therapeutics and possibly to inform personalized medicine. In the genomics field for sporadic diseases, which accounts for 90% of ALS patients, larger GWAS studies can allow researchers to calculate polygenic risk scores (PRS). These scores can be used to determine which pathways may be driving disease risk or to calculate a genetic risk for disease in a given individual. PRS can be calculated using the summary statistics from massive association studies (i.e. GWAS) and therefore can be updated with every new association study that is released [208, 209]. In the future, it may be possible to use these scores in the same way that we use current genetic testing, but rather than looking at one or a few genes, the whole genome will be considered [210]. While some PRS scores are currently being used for melanoma [211], coronary artery disease [212] and diabetes [213], PRS is still primarily in the research phase for ALS and many other diseases. One PRS has been completed recently in ALS but did not seem likely to have clinical utility based on the small proportion of heritability that it could explain [214]. Perhaps this is because ALS is driven by variation other than common SNPs, so PRS calculated from GWAS may never be sufficient [107]. Possibly some of the long-read sequencing and multi-omic approaches can be utilized to improve the predictive power of PRS. It should be noted that PRS will require large datasets for training and is highly dependent on population structure [215]. Because most GWAS have been completed in Caucasian/European populations, there is a risk that if PRS are introduced in the clinic, they may not benefit diverse populations worsening current disparities in healthcare [215]. Therefore, efforts should continue to be made to include patients of many genetic population backgrounds in sequencing studies.

## Conclusions

Classical gene discovery methods have helped to uncover important genetic variation that is causative or modulates risk of developing ALS. Linkage analyses in familial studies and Sanger sequencing will continue to remain pertinent to identify variants in known genomic regions. Newer sequencing methods have facilitated discovery of pathogenic variation in individuals, families and even in large populations not just for known genes, but across the entire genome. The emergence of long-read sequencing has shed light on more complex variation, including repeat expansions and other types of structural variation in ALS. In the upcoming years, we expect that long-read sequencing technologies will continue to be used by more researchers and clinicians, especially if the costs decrease, as it provides an unbiased approach to capture the complex genetics of ALS. Integration of multiple methods using multi-omic techniques to determine the effect of variants will also continue to help nominate genes and pathways that contribute to disease pathogenesis. Improving our understanding of the origin and course of the disease will be useful not only in developing hypotheses for research but will be equally important clinically to help with genetic testing and disease prediction, ultimately offering therapeutic solutions for this devastating disease.

## Data Availability

Not applicable.

## Abbreviations

ALS: Amyotrophic lateral sclerosis
ATAC-seq: Assay for transposase accessible chromatin sequencing
CCS: Circular consensus sequence
CHIP-seq: Chromatin immunoprecipitation sequencing
CLR: Continuous long read
CRISPR: Clustered regularly interspaced short palindromic repeats
eQTL: Expression quantitative trait locus
fALS: Familial ALS
FTD: Frontotemporal dementia
GWAS: Genome-wide association study
HiFi: High fidelity
iPSC: Induced pluripotent stem cell
meQTL: Methylation quantitative trait locus
MND: Motor neuron disease
No-Amp: No amplification
ONT: Oxford Nanopore Technologies
PacBio: Pacific Biosciences
pQTL: Protein quantitative trait locus
PRS: Polygenic risk score
QTL: Quantitative trait locus
RNAseq: RNA sequencing
sALS: Sporadic ALS
SCA: Spinocerebellar ataxia
SMRT sequencing: Single-molecule real-time sequencing
SNP: Single nucleotide polymorphism
SNV: Single nucleotide variant
TWAS: Transcriptome-wide association study
WES: Whole-exome sequencing
WGS: Whole-genome sequencing
ZMW: Zero mode waveguide
